# Topical Isotretinoin: Current Applications, Challenges, and Future Directions

**DOI:** 10.7759/cureus.90820

**Published:** 2025-08-23

**Authors:** Iman Bouchelkia, Stephen Tyring

**Affiliations:** 1 Biomedical Sciences, Tilman J. Fertitta Family College of Medicine, Houston, USA; 2 Dermatology, The University of Texas Health Science Center, Houston, USA

**Keywords:** dermatology, retinoid therapy, skin barrier, topical isotretinoin, topical retinoid efficacy

## Abstract

Topical isotretinoin is designed to work locally on the skin with limited systemic absorption and is being explored as a potential alternative to more commonly used topical retinoids. However, limited research, formulation challenges, and regulatory hurdles have slowed its path to widespread use. It appears to perform similarly to other topical retinoids and may offer added benefits such as improved tolerability and greater formulation stability. Additionally, its ability to integrate into combination therapies may further enhance its clinical utility. Despite these benefits, the lack of extensive randomized controlled trials limits understanding of its optimal use, long-term effects, and safety profile. Future research is essential to determine its role in dermatologic treatment, but its potential to expand therapeutic options remains promising.

## Introduction and background

Topical isotretinoin is a 13-cis-retinoic acid designed to regulate keratinocytes and sebaceous gland modulation and has anti-inflammatory effects [1]. Unlike oral isotretinoin, which is widely used for severe acne, topical isotretinoin remains largely experimental, with limited availability and most studies confined to clinical trials. Despite its promising potential for localized efficacy with fewer systemic side effects, its clinical adoption has been slow due to regulatory hurdles, formulation challenges, and limited large-scale studies. The aim of this narrative review is to summarize the current evidence on topical isotretinoin, including its mechanism of action, therapeutic applications, and reported clinical outcomes.

## Review

Mechanism of action

Oral isotretinoin has been found to be the only therapy that impacts all of the major etiological factors implicated in acne [2]. It achieves this by influencing cell cycle progression, differentiation, and apoptosis, which in turn result in significant sebum reduction, affect comedogenesis, and reduce *Propionibacterium acnes* in the skin [2]. Studies have shown that isotretinoin directly induces apoptosis within sebaceous glands, a mechanism that contributes to reduced sebum production independent of receptor signaling [3]. Topical isotretinoin also targets multiple major acne pathophysiology factors, including keratinization, sebaceous gland activity, and inflammation, but its effects are localized and milder due to minimal systemic absorption. Due to its confined impact, the topical formulation offers a targeted approach associated with reduced systemic exposure and a lower incidence of systemic side effects. This makes it a potential alternative for milder cases in patients who are unable to tolerate systemic retinoids.

In contrast, tretinoin, an all-trans retinoic acid, mediates its effects by binding retinoic acid and retinoid X receptors by blocking inflammatory mediators [4]. As a result, this causes modification of excessive keratinization of the epithelial cells, promoting cellular turnover and shedding. While tretinoin effectively modifies excessive keratinization and promotes cellular shedding, its impact on sebum production is minimal. Approximately 80% of topically applied tretinoin remains on the skin surface, limiting its influence on deeper sebaceous gland activity [5]. 

Clinical applications and efficacy 

Although limited in availability and primarily studied in clinical trials, topical isotretinoin has demonstrated potential as a localized treatment, offering a targeted approach with fewer systemic side effects compared to its oral counterpart. While oral isotretinoin remains the gold standard for severe acne, the topical formulation provides an alternative for mild to moderate cases, particularly in patients who cannot tolerate systemic retinoids.

Congenital Icthyosis

In a recent randomized CONTROL study, a novel topical isotretinoin formulation, TMB-001, was found to be more effective than vehicle in treating congenital ichthyosis subtypes (autosomal recessive lamellar ichthyosis (ARCI-LI) and X-linked recessive ichthyosis (XLRI)). This phase IIb CONTROL study found that TMB-001 significantly improved scaling scores (p = 0.03) and was well-tolerated, with most adverse events being localized application-site reactions [6]. These results suggest that TMB-001 may offer a promising non-systemic treatment option for congenital ichthyosis. When analyzing the identifying characteristics of congenital ichthyosis patients who responded to treatment with TMB-001, the study found that participants with a higher Dermatology Life Quality Index (DLQI) and Itch-Numeric Rating Scale (I-NRS) scores and less extensive disease had better treatment outcomes [7]. Interestingly, TMB-001 0.05% performed better than 0.1%, which was unexpected [8]. Additionally, the safety and tolerability profile of TMB-001 remained consistent with phase IIa adverse events and with the known safety profile of topical retinoids [8]. These findings could help identify which patients may benefit most from topical isotretinoin therapy as an alternative to oral retinoids for congenital ichthyosis.

Acne Vulgaris

Topical isotretinoin has also been investigated as a treatment for acne vulgaris, offering a potential alternative to other topical retinoids. A prospective, randomized clinical trial comparing topical isotretinoin 0.05% gel vs. topical retinoic acid 0.05% cream found that both treatments significantly reduced inflammatory and noninflammatory lesions over 12 weeks, with most patients improving from grades III/IV to grades I/II based on the Plewig and Kligman acne grading system [9]. However, topical isotretinoin demonstrated superior tolerability, with fewer patients experiencing adverse effects such as stinging, erythema, and desquamation compared to retinoic acid (47% vs. 67%) [9]. These results suggest that topical isotretinoin may serve as a viable alternative to tretinoin, particularly for patients with sensitive skin or poor tolerance to traditional topical retinoids.

Topical isotretinoin has also been studied against benzoyl peroxide, a well-established acne treatment. In a double-blind, randomized trial of 77 patients with mild to moderate acne, both topical isotretinoin 0.05% and benzoyl peroxide 5% significantly reduced noninflammatory lesions at weeks four, eight, and 12 (p < 0.01), with both treatments causing mild, well-tolerated irritation [10]. Both treatments caused modest, well-tolerated discomfort. Topical isotretinoin seems to target comedogenesis, which makes it helpful for comedonal acne, although benzoyl peroxide showed quicker effectiveness for inflammatory acne.

Given the comparable efficacy of isotretinoin and other topical acne treatments, its stability under different light conditions may also influence treatment outcomes. A study comparing 0.05% isotretinoin and 0.05% tretinoin found that tretinoin degrades more rapidly under fluorescent light, with only 25% remaining intact after two hours, while isotretinoin retained 60% of its original form [11]. When comparing these effects in moderate acne, there was no significant difference found in lesion reduction. These findings suggest that light exposure significantly impacts tretinoin’s bioavailability, which may explain variability in clinical outcomes.

Photodamage

Beyond acne treatment, topical isotretinoin has also been explored for its potential in addressing photodamaged skin. In a recent placebo-controlled trial, 0.1% isotretinoin was evaluated in 800 patients with moderate to severe photodamage, showing significant clinical improvements in wrinkling, skin texture, and hyperpigmentation (p < 0.01) [12]. Patient-reported outcomes aligned with physician assessments, with 35% of isotretinoin users reporting marked improvement compared to 15% in the vehicle group [12]. Localized irritation was observed; however, it was mostly mild to moderate, with a low percentage of patients discontinuing the treatment. Additionally, minimal systemic absorption of retinoid was observed, indicated by stable plasma retinoid levels.

Actinic Keratoses (AKs)

Isotretinoin has also been investigated for its potential in treating AKs, a precancerous condition caused by chronic sun exposure. In a randomized, placebo-controlled trial, 0.1% isotretinoin cream applied twice daily for 24 weeks significantly reduced facial AKs compared to placebo (p = 0.001), with 66% of isotretinoin-treated patients achieving a >30% lesion reduction [13]. Isotretinoin may still be helpful in managing sun damage over the long run, although it cannot match the more rapid AK therapies.

Safety and side effects 

Although topical isotretinoin is thought to have fewer systemic adverse effects than oral isotretinoin, new research indicates that it may still aggravate the surface of the eyes and cause symptoms of dry eyes. In a study of 43 patients using topical isotretinoin and erythromycin (Isotrexin gel) once daily, mean tear osmolarity increased from 282.09 ± 8.95 mOsm/L to 300.39 ± 16.65 mOsm/L (p < 0.001), indicating worsening tear film quality [14]. Fluorescein break-up time (BUT) decreased significantly from 11.93 ± 1.12s to 6.65 ± 3.03s (p < 0.001), while the OSDI score increased from 5.41 ± 3.65 to 21.53 ± 12.95 (p < 0.001), indicating significant dry eye symptoms, with punctate epitheliopathy observed in 51% of eyes, suggesting corneal surface damage [14]. Given these results, doctors should recommend routine ophthalmologic monitoring in symptomatic individuals and screen acne patients for pre-existing dry eye symptoms prior to applying topical isotretinoin. To improve tolerability and treatment compliance, ocular irritation may be lessened by using artificial tears or modifying the frequency of applications.

A key concern with retinoids is their teratogenic potential, particularly with oral isotretinoin, which has strict pregnancy restrictions due to its high systemic absorption. However, a study evaluating topical isotretinoin (0.1% cream) applied daily over 2,300 cm² for 42 days found that plasma isotretinoin levels increased by only 48% ± 9.2% and 4-oxo-isotretinoin by 77% ± 13% (p < 0.001), far lower than the increases seen with a standard 5,000 IU daily vitamin A supplement (141% ± 19% and 171% ± 27%, respectively) [15]. Tretinoin levels remained unchanged, suggesting minimal systemic conversion. These results indicate that topical isotretinoin has negligible systemic absorption, reducing concerns about teratogenicity. However, precaution is still advised in pregnant individuals, as additional long-term studies are needed to confirm absolute safety.

Discussion 

Topical retinoids are a staple of dermatological therapy, often used for their comedolytic, anti-inflammatory, and skin-turnover properties [16]. Although adapalene and tretinoin have been well-researched and used in clinical settings, topical isotretinoin is still a relatively new therapy with potential benefits such as less systemic absorption and focused effectiveness. This targeted effect helps reduce sebum production without damaging the surrounding skin. The potential of topical isotretinoin extends beyond its current experimental status, offering a new perspective on how retinoids can be used in dermatology. Its ability to improve skin texture, reduce scaling, and enhance epidermal renewal suggests that it could be beneficial for a larger patient population, including those with chronic inflammatory or hyperproliferative conditions.

One of the most promising aspects of topical isotretinoin is its potential for long-term skin maintenance and disease prevention. Its effects on photoaging and AKs suggest it could be explored as a preventative option for high-risk individuals, such as those with extensive sun exposure or precancerous lesions. This could place it in a new category of dermatologic interventions, where its function extends beyond treatment and into proactive skin health management. Additionally, two crucial facets of dermatological treatment - patient adherence and tolerability - may improve with topical isotretinoin. Many patients discontinue topical retinoids due to irritation, dryness, or poor tolerability, especially those with sensitive skin or chronic conditions [17]. For those who suffer from the negative effects of other retinoids, isotretinoin formulations may offer an option if they continue to show similar effectiveness with better tolerability. For example, its potential for combination therapy with other substances, such as anti-inflammatory chemicals or benzoyl peroxide, may also increase its efficacy while reducing irritation, expanding its use for a wider range of dermatological diseases. Benzoyl peroxide is currently the preferred topical antimicrobial because it minimizes the risk of resistance development, making it a reliable option for long-term use. It has been shown to reduce *Cutibacterium acnes* concentrations and suppress inflammation. Its strong oxidative properties can lead to a 90% reduction in* Cutibacterium acnes *within seven days, and no bacterial resistance to benzoyl peroxide has been reported to date [18-20].

Moreover, the absence of extensive randomized controlled trials restricts the capacity to specify ideal treatment regimens, therapeutic length, and practical effectiveness. Most existing studies have been relatively short in duration, making it unclear whether long-term use maintains efficacy or leads to diminishing returns. Without long-term data, it remains uncertain how frequently patients would need continued application, whether intermittent therapy could be effective, or if prolonged use poses any unforeseen safety risks.

To fully integrate topical isotretinoin into clinical practice, future research must address these gaps. Conducting well-powered, long-term, randomized controlled trials will be essential for defining optimal treatment regimens, duration of therapy, and safety over time. Until these questions are answered, its role in dermatologic treatment remains promising but not yet fully established.

## Conclusions

In dermatology, topical isotretinoin is at an intriguing crossroads: it has great potential in principle, but has not yet reached its full potential in practice. It is being explored for use in conditions such as acne, keratinization disorders, and photodamage, but its wider adoption remains limited due to unanswered questions about long-term safety and regulatory approval. Its distinct qualities, including minimal systemic absorption and potential for enhanced tolerability, make it a desirable substitute for other retinoids, particularly for individuals who have trouble with conventional therapies. Its position is still one of promise rather than certainty, though, until more extensive, well-planned trials define its ideal application, safety profile, and duration of effects. Topical isotretinoin has the potential to revolutionize retinoid therapy by bridging the gap between accessibility, tolerability, and efficacy in dermatological care with further study. 
